# Clinical and microbiological characteristics of neonates with bacteremia by NDM-producing *Klebsiella pneumoniae* in a Peruvian referral hospital

**DOI:** 10.17843/rpmesp.2026.432.15600

**Published:** 2026-06-04

**Authors:** Flavia Lara-Herrera, Ana Amable-Castilla, Daniela Mori-Rodriguez, Giancarlo Pérez-Lazo

**Affiliations:** 1 Facultad de Medicina Humana, Universidad de Piura, Lima, Peru.; 2 Hospital Nacional Guillermo Almenara Irigoyen, Lima, Peru.

**Keywords:** Newborn, Bacteremia, Klebsiella pneumoniae, Carbapenem-Resistant *Enterobacteriaceae*, Peru

## Abstract

We conducted a descriptive cross-sectional study in the neonatology service of the Hospital Nacional Guillermo Almenara Irigoyen (2020-2024) to characterize the clinical and microbiological profile of neonates with carbapenemase-producing *Klebsiella pneumoniae* bacteremia. Nineteen neonates were included; most were preterm and low birth weight, and approximately half presented bacteremia secondary to a respiratory focus. All strains carried the *bla*_NDM_ gene. *In vitro*, 84.2% (16/19) were susceptible to amikacin and 40% (6/15) to tigecycline. All isolates had intermediate susceptibility to colistin and resistance to multiple antimicrobials. Empirical therapy was appropriate in 7/16 (43.8%) and about a fifth of the patients died. The identification of *bla*_NDM_ reinforces the importance of local genomic surveillance to guide empirical therapy. The type of carbapenemase and its impact on mortality is a future research gap.

## INTRODUCTION

Infections by carbapenemase-producing Enterobacterales (CPE) represent a growing threat to public health. These infections double the risk of mortality in the general population and quintuple it in neonates, who constitute a vulnerable group ^1^^,^^2^. Class A (KPC), B or metallo-β-lactamase (NDM, VIM, IMP), and D (OXA-48 and its derivatives) carbapenemases, have a high level of antibiotic resistance ^3^. Among CPE, *Klebsiella pneumoniae* (KP) is associated with severe nosocomial infections, and is considered a critical priority pathogen according to the World Health Organization ^4^.

The distribution of carbapenemases in neonates varies by region. In China, NDM-1 predominates in neonatal sepsis ^5^, a finding corroborated by a global meta-analysis in neonates infected by carbapenemase-producing *Klebsiella pneumoniae* (KpPC), in whom 638 NDM-1-producing strains were isolated in 11 countries, followed by OXA-48 and KPC ^6^. In Portugal, the KPC genotype predominated with 57% of colonized neonates ^7^. In South America, data are limited. Colombia documented four cases of bacteremia by NDM-1-producing KP and two cases of colonization ^8^. In Brazil, five cases of colonization by KPC-type KpPC were reported; and in Venezuela, 12 cases of bacteremia and two of colonization with co-production of KPC and VIM carbapenemases were reported ^9^^,^^10^. In Peru, a study identified two neonates with carbapenem-resistant *Klebsiella pneumoniae* (KpRC) isolates in blood cultures, which represented 7.7% of the strains obtained in that age group ^11^.

In Peru, the clinical impact of KpPC is unknown, so the contribution of this initial exploration can guide therapy, as well as the development of specific prevention and control strategies. Therefore, our objective was to describe the clinical and microbiological characteristics of neonates with bacteremia due to *Klebsiella pneumoniae* carbapenemase-producing attended at the Hospital Nacional Guillermo Almenara Irigoyen (HNGAI).

**KEY MESSAGES**

Motivation for the study. Infections caused by carbapenemase-producing *Klebsiella pneumoniae* are associated with high neonatal mortality. In Peru, clinical and microbiological information on this infection is scarce, especially in neonates.

Main findings. Most neonates were premature, had low birth weight, and required multiple invasive support. Isolates showed multidrug resistance, empirical treatment was inappropriate in more than half of the cases, and nearly one-fifth died.

Public health implications. It is a priority to reinforce epidemiological surveillance and infection control programs in neonatal units to contain the dissemination of multidrug-resistant strains and improve neonatal survival.

## THE STUDY

### Design and study setting

Descriptive cross-sectional study conducted in the Neonatology service of the HNGAI during the 2020-2024 period. This hospital is a high-complexity facility of the Social Security system and is a national reference center located in Lima, Peru.

### Participants

**Figure 1:**
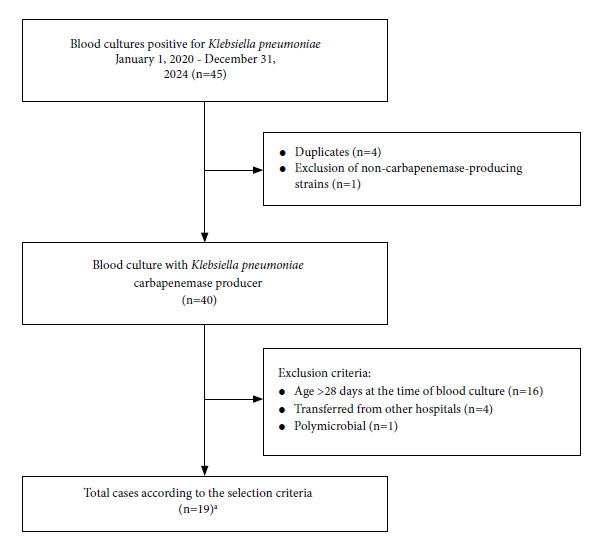
Flowchart of included and excluded neonates with positive blood culture for carbapenemase-producing *Klebsiella pneumoniae*.

^a^ The microbiological and antimicrobial susceptibility analysis presented in the study exclusively corresponds to the strains from the included patients (n=19).

The population included neonates hospitalized in the neonatology area or the Neonatal Intensive Care Unit (January 1, 2020 - December 31, 2024), with positive monomicrobial blood culture for KpPC within the first 28 days of life. Classification into primary or secondary bacteremia was based on the clinical origin of the infection and not on the type of microbiological sample. The selection flowchart is shown in figure 1.

### Variables of study

We recorded sex, birth weight (in grams), gestational age (in complete weeks), type of delivery, and the dates of birth, blood culture collection, and hospital discharge. Age at bacteremia was calculated in days as the difference between the blood culture collection date and birth date. Prematurity was defined as birth before 37 weeks of gestation, and low birth weight as < 2500 grams. An infectious disease specialist (GPL) classified bacteremias as primary or secondary based on medical records.

The total length of hospital stay in days was calculated as the difference between the outcome date (alive or deceased) and admission. Also determined were the length of stay until KpPC isolation (in days) and the time until appropriate targeted antibiotic therapy administration (in hours), determined by the *in vitro* susceptibility to at least one antibiotic ^12^^,^^13^. The susceptibility profile, the empirical therapy administered, the adequacy of said therapy based on antibiogram results, and the definitive treatment of each neonate were recorded.

### Microbiological Procedures

Microbiological data were requested from the microbiology department of HNGAI and obtained from electronic medical records. At HNGAI, the initial identification of blood cultures was performed by conventional microbiology. Antimicrobial susceptibility was performed using the VITEK-2® GN platform and carbapenemase confirmation using immunochromatographic methods (RESIST-5 O.K.N.V., Coris Bio-Concept) and Xpert ® Carba (Cepheid). Colistin susceptibility testing was performed using the Colistin agar spot testagar. The minimum inhibitory concentration of each antibiotic followed the guidelines established by the Clinical and Laboratory Standards Institute (CLSI). For colistin, the current CLSI breakpoints (M100) were used, which consider only the categories: intermediate (≤2 µg/mL) and resistant (≥4 µg/mL) for Enterobacterales ^14^.

### Statistical Analysis

We report the measures of central tendency (median, interquartile range [IQR]) for quantitative variables, and frequency for qualitative variables. Descriptive analysis was performed using the Jamovi software (version 2.3.28).

### Ethical aspects

The protocol was approved by the Ethics Committee of the Universidad de Piura (No. T0624-11) and the Institutional Ethics Committee for Research of HNGAI (No. 108-2024).

## FINDINGS

From a total of 40 blood cultures with KpPC isolation identified during the study period, 19 corresponded to neonates within the first 28 days of life, according to the population defined in the study, these being the cases finally analyzed (figure 1). All were NDM-producing strains; no other carbapenemases were identified. 52.6% were females, 94.7% were premature, and all had low birth weight. The median gestational age was 27 weeks (IQR: 26 - 30). 94.7% (18/19) had secondary bacteremia. Support therapy included the use of an orogastric tube and central catheter in all 19 cases, parenteral nutrition in 18 (94.7%), and mechanical ventilation in 15 cases (78.9%). All neonates had some type of metabolic alteration. 38.9% of neonates (7/19) had two metabolic alterations simultaneously. 84.2% (16/19) of neonates presented four or more comorbidities (table 1).

**Table 1:** Clinical characteristics, therapeutic support and comorbidities of neonates with bacteremia due to *Klebsiella pneumoniae* NDM-producing (n=19).

Variables		Absolute Frequency (n=19)	Percentage (%) ^b^
Sex			
	Female	10	52.6
	Male	9	47.4
Mode of delivery			
	Natural	2	10.5
	Cesarean	17	89.5
	Prematurity (yes)	18	94.7
	Birth weight (low<2500 g)	19	100.0
Area at diagnosis			
	ICU	15	78.9
	Hospitalization	4	21.1
Type of bacteremia			
	Primary	1	5.3
	Secondary	18	94.7
Focus of infection^a^			
	Respiratory origin	9	50.0
	Gastrointestinal origin	7	38.9
	Catheter-related	2	11.1
Supportive therapy^b^			
	Orogastric tube	19	100.0
	Central catheter	19	100.0
	Total parenteral nutrition	18	94.7
	Mechanical ventilation	15	78.9
	Use of surfactant	11	57.9
	Use of vasopressors	8	42.1
	Use of corticosteroids	6	31.6
	Urinary catheter	5	26.3
	Surgeries	1	5.3
	Peripheral catheter	1	5.3
Comorbidities			
	Metabolic Alterations^c^	19	100.0
	Respiratory distress	17	89.5
	Hyaline membrane disease	14	73.7
	Anemia	11	57.9
	Hyperglycemia	10	52.6
	Hypoalbuminemia	6	31.6
	Hypoglycemia	6	31.6
	Congenital heart disease	4	21.1
	Renal failure	1	5.3
	Other comorbidity^d^	14	73.7
Number of comorbidities per neonate			
	One	0	0.0
	Two	1	5.3
	Three	2	10.5
	Four or more	16	84.2
Final condition			
	Live	15	78.9
	Deceased	4	21.1

ICU: intensive care unit; g: grams.

a The focus of infection is described only in cases of secondary bacteremia (n=18); percentages were calculated on this subgroup.

b The percentage is calculated considering as the denominator the 19 neonates.

c Metabolic acidosis: 47.4% (9/19); hypocalcemia: 47.4% (9/19); hyponatremia: 42.1% (8/19); respiratory acidosis: 21.1% (4/19); hypernatremia: 15.8% (3/19).

d Neonatal jaundice: 57.1% (8/14); chorioamnionitis: 14.3% (2/14); necrotizing enterocolitis: 7.1% (1/14).

The median hospital stay was 82 days (IQR: 30.5 - 92.5), the median time to blood culture collection was 9 days (IQR: 7.50 - 15.5), and the median time to initiation of appropriate targeted antibiotic therapy was 94.5 hours (IQR: 48.3 - 103). Four of the 19 neonates died during the entire hospitalization (21.1%), of whom three died within the first 28 days of birth (15.8%). In these latter, the median days from bacteremia to death was 2 (IQR: 1.5 - 6.5).

**Figure 2:**
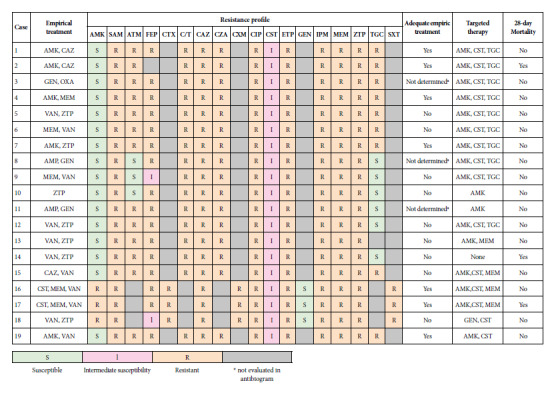
Individual empirical and definitive antibiotic therapy and resistance profile of neonates with NDM-producing *K. pneumoniae* bacteremia.

Empirical antimicrobial treatment included regimens with piperacillin/tazobactam in 36.8% (7/19) and vancomycin in 57.9% (11/19). The combinations used empirically were piperacillin/tazobactam and vancomycin in 26.3% (5/19), followed by ceftazidime and amikacin in 11.1% (2/19). Empirical treatment was appropriate in 7 of 16 cases (43.8%); additionally, it was not possible to define adequacy in three cases where ampicillin, oxacillin, and gentamicin were used because they were not evaluated in the antibiograms. Susceptibility was preserved to colistin, amikacin, and tigecycline in 100% (19/19), 84.2% (16/19), and 40% (6/15) of the isolates, respectively. Resistance was observed to ampicillin/sulbactam, ceftazidime, ciprofloxacin, meropenem, imipenem, and piperacillin/tazobactam in all cases. Directed antimicrobial therapy was administered in 18 neonates; of these, 16 (88.9%) received combined regimens, with the combination of amikacin, colistin, and tigecycline being the most frequent (figure 2).

S: susceptible; I: intermediate; R: resistant; ATM: aztreonam; AMK: amikacin; AMC: amoxicillin/clavulanic acid; AMP: ampicillin; SAM: ampicillin/sulbactam; ATM; az-treonam; FEP: cefepime; CTX: cefotaxime; C/T: ceftolozane/tazobactam; CAZ: ceftazidime; CZA: ceftazidime/avibactam; CXM: cefuroxime; CIP: ciprofloxacin; CST: colistin; ETP: ertape-nem; GEN: gentamicin; IPM: imipenem; MEM: meropenem; TGC: tigecycline; SXT: trimethoprim/sulfamethoxazole; OXA: oxacillin; VAN: vancomycin; ZTP: piperacillin/tazobactam. ^a^ It was not possible to determine the adequacy of empirical therapy in cases 3, 8, and 11 due to the lack of evaluation of ampicillin, oxacillin, and gentamicin in the respective antibiograms.

## DISCUSSION

We found that all isolated *KpPC* strains were NDM producers. Clinically, the neonates were preterm and had low birth weight. The focus of infection in half of the cases was respiratory. Microbiologically, the strains were susceptible to amikacin and tigecycline, and showed intermediate susceptibility to colistin, while all strains were resistant to ampicillin/sulbactam, ceftazidime, ciprofloxacin, meropenem, imipenem, and piperacillin/tazobactam. With this profile, empirical treatment was appropriate in seven of sixteen neonates, and one-fifth of the neonates died.

In our sample, only the *bla*_NDM_ gene was identified, a dominant carbapenemase in 14 countries worldwide ^6^. However, certain regions show different profiles, with KPC-2 being the most frequent type in a pediatric hospital in Brazil, while in a Spanish cohort of children, VIM predominated ^9^^,^^15^.

Regarding the susceptibility profile, we found similarity with a study in neonates from a hospital in China, in whom KpRC with sensitivity to colistin and tigecycline was found in most isolates; however, unlike our study, most neonates presented resistance to amikacin ^16^. Likewise, tigecycline resistance in our study (60%) was higher than that reported in children under 5 years of age with sepsis by *K. pneumoniae* in Ethiopia (39%) ^17^. This finding highlights the global relevance of this resistance profile and the influence of epidemiological factors on its variability.

The use of colistin and tigecycline as definitive combined treatment was frequent in most neonates; however, optimal regimens for these antibiotics in this population have limited evidence ^18^. Empirical therapy was appropriate in 43.8% of neonates, which may be a result of the heterogeneity in the treatment regimens provided. The time from blood culture collection until initiation of targeted therapy had a median of 95 hours, which reflects the difficulties in early optimization of antibiotic therapy against NDM and could be related to the mortality found in our study. Evidence shows that appropriate targeted therapy reduces mortality by 67% in children with KpRC bacteremia ^16^. Furthermore, timely initiation of an antibiotic with confirmed *in vitro* activity reduces 30-day mortality by 80% ^15^.

Mortality in our study was ~21%, this result is consistent with what was estimated in a systematic review for neonatal KpRC (pooled mortality ~23%), in this study the combination of high host vulnerability and late therapeutic windows were determinants of the outcome ^6^. However, in a study in China they reported a higher mortality in neonates with KpRC bacteremia (39.4%) ^16^. The lower mortality in our study could be related to the conserved susceptibility of antimicrobials such as colistin and amikacin, as well as the severity of the cases.

Furthermore, the observed mortality could be explained by the clinical characteristics of our neonates: prematurity, low birth weight, multiple invasive support, respiratory or gastrointestinal foci, and prolonged hospitalization. These findings are consistent with studies in China and Egypt, where prematurity, low birth weight, and the use of invasive devices, including total parenteral nutrition, were associated with an increased risk of infection by carbapenem-resistant Enterobacterales (CRE) ^19^^,^^20^.

The most frequent infection foci were respiratory, followed by gastrointestinal. These results contrast with a systematic review conducted in Latin America in pediatric patients with bloodstream infection by CRE, who reported that 64% of bloodstream infections by CPE were secondary to an intra-abdominal focus, followed by cutaneous foci at 32% ^21^. This contrast may be due to the high frequency of mechanical ventilation in our population (78%).

Prolonged length of stay reflects a greater risk of nosocomial infections and comorbidities. In our study, the median hospital stay was 82 days, higher than reported in Italy where a median of 70 days was documented among neonates with multidrug-resistant KP bacteremia ^22^, while in China, 36 days were recorded in neonates with KpRC bacteremia ^16^. This difference could reflect an earlier diagnosis or a faster progression of the infection in our population. Additionally, we reported a median age to bacteremia of 9 days, which is lower than reported in Portugal and Brazil, which was 12 days in cases of CPE colonization ^7^^,^^9^. These findings suggest a possible earlier colonization in our sample that could be due to individual and hospital environment factors.

Among the limitations, this study was retrospective and had a reduced sample size; however, we collected information from all neonates with KpPC NDM-type bacteremia treated under usual conditions at the same reference institution. Furthermore, the definition of appropriate empirical antimicrobial therapy could be affected by partial susceptibility panels; despite this, we described its adequacy in 16 neonates empirically treated with antibiotics and evaluated with the susceptibility profile. Also, the therapeutic regimens observed reflect clinical practice in a public reference hospital and were conditioned by the local availability of antimicrobials. In infections caused by metallo-β-lactamase-producing enterobacteria, regimens such as the combination of ceftazidime-avibactam plus aztreonam have generated interest; however, their use in neonates still lacks robust clinical evidence. Furthermore, the use of ceftazidime-avibactam in neonates has historically been off-label, given that its initial regulatory approval was restricted to patients older than 3 months ^23^^,^^24^

Another limitation was the absence of molecular typing studies or clonality tests of the isolates. Although all strains were NDM producers, clonal relatedness was not determined by any genomic sequencing technique. Therefore, it is not possible to determine if the cases correspond to the dissemination of a single clone within the neonatal unit or to multiple independent introductions. This information would be relevant to better understand the transmission dynamics and guide infection control strategies.

In conclusion, NDM-type KpPC predominated in low-birth-weight and premature neonates from the neonatal ICU service. Additionally, we observed a higher frequency of secondary bacteremia with a respiratory focus. This study evidenced that NDM-producing *Klebsiella pneumoniae* was resistant to multiple drugs; however, it was susceptible to amikacin and colistin. The identification of *bla*_NDM_ reinforces the importance of local genomic surveillance to guide empirical therapy. The type of carbapenemase and its impact on mortality represent a future research gap.
