# Supplementary material for: Clinical and microbiological characteristics of neonates with bacteremia by NDM-producing Klebsiella pneumoniae in a Peruvian referral hospital
Source: Rev Peru Med Exp Salud Publica. 2026 Jun 4;43(2):217–23. doi: 10.17843/rpmesp.2026.432.15600 (PMC13456927; doi:10.17843/rpmesp.2026.432.15600)
Supplement: Translation

# Translation: Características clínicas y microbiológicas de neonatos con bacteriemia por *Klebsiella pneumoniae* productora de NDM en un hospital peruano de referencia

**Authors:** Flavia Lara-Herrera, Ana Amable-Castilla, Daniela Mori-Rodriguez, Giancarlo Pérez-Lazo

**Affiliations:** 1 Facultad de Medicina Humana, Universidad de Piura, Lima, Perú.; 2 Hospital Nacional Guillermo Almenara Irigoyen, Lima, Perú.

**Keywords:** Neonatos, Bacteremia, Klebsiella pneumoniae, *Enterobacteriaceae* Resistentes a los Carbapenémicos, Perú

## Abstract

Realizamos un estudio transversal descriptivo en el servicio de neonatología del Hospital Nacional Guillermo Almenara Irigoyen (2020-2024) para caracterizar el perfil clínico y microbiológico de neonatos con bacteriemia por *Klebsiella pneumoniae* productora de carbapenemasa. Se incluyeron 19 neonatos; la mayoría fue pretérmino y de bajo peso al nacer, y aproximadamente la mitad presentó bacteriemia secundaria a foco respiratorio. Todas las cepas portaban el gen *bla*_NDM_. *In vitro*, 84,2% (16/19) fueron susceptibles a amikacina y 40% (6/15) a tigeciclina. Todos los aislamientos tuvieron susceptibilidad intermedia a colistina y resistencia a múltiples antimicrobianos. La terapia empírica fue apropiada en 7/16 (43,8%) y cerca de una quinta parte de los pacientes falleció. La identificación de *bla*_NDM_ refuerza la importancia de la vigilancia genómica local para orientar la terapia empírica. El tipo de carbapenemasa y su impacto en la mortalidad es una brecha de investigación futura.

## INTRODUCCIÓN

Las infecciones por Enterobacterales productores de carbapenemasas (EPC) representan una amenaza creciente para la salud pública. Estas infecciones duplican el riesgo de mortalidad en la población general y lo quintuplican en neonatos, quienes constituyen un grupo vulnerable ^1^^,^^2^. Las carbapenemasas de clase A (KPC), B o metalo-β-lactamasas (NDM, VIM, IMP), y D (OXA-48 y sus derivados), tienen un elevado nivel de resistencia a antibióticos ^3^. Entre los EPC, *Klebsiella pneumoniae* (KP) está asociada a infecciones nosocomiales graves, y es considerada un patógeno prioritario crítico según la Organización Mundial de la Salud ^4^.

La distribución de carbapenemasas en neonatos varía según la región. En China, NDM-1 predomina en sepsis neonatal ^5^, hallazgo corroborado por un metaanálisis global en neonatos infectados por *Klebsiella pneumoniae* productora de carbapenemasa (KpPC), en quienes se aisló 638 cepas productoras de NDM-1 en 11 países, seguido de OXA-48 y KPC ^6^. En Portugal predominó el genotipo KPC con un 57% de neonatos colonizados ^7^. En Sudamérica, los datos son limitados. Colombia documentó cuatro casos de bacteriemia por KP productora de NDM-1 y dos casos de colonización ^8^. En Brasil, fueron notificados cinco casos de colonización por KpPC tipo KPC; y en Venezuela fueron reportados 12 casos de bacteriemia y dos de colonización con co-producción de carbapenemasas KPC y VIM ^9^^,^^10^. En Perú, un estudio identificó dos neonatos con aislamientos de *Klebsiella pneumoniae* resistentes a carbapenémicos (KpRC) en hemocultivos, lo que representó el 7,7% de las cepas obtenidas en ese grupo etario ^11^. 

En Perú se desconoce el impacto clínico de KpPC, por lo que el aporte de esta exploración inicial puede guiar a la terapéutica, así como el desarrollo de estrategias específicas de prevención y control. Por lo tanto, nuestro objetivo fue describir las características clínicas y microbiológicas de neonatos con bacteriemia por *Klebsiella pneumoniae* productora de carbapenemasa atendidos en el Hospital Nacional Guillermo Almenara Irigoyen (HNGAI).

**MENSAJES CLAVE**

Motivación para realizar el estudio. Las infecciones por *Klebsiella pneumoniae* productora de carbapenemasas se asocian a elevada mortalidad neonatal. En Perú, la información clínica y microbiológica sobre esta infección es escasa, especialmente en neonatos.

Principales hallazgos. La mayoría de neonatos fueron prematuros, de bajo peso al nacer y con necesidad de soporte invasivo múltiple. Los aislamientos presentaron multirresistencia, el tratamiento empírico fue inapropiado en más de la mitad de los casos y cerca de una quinta parte falleció.

Implicancias para la salud pública. Es prioritario reforzar la vigilancia epidemiológica y los programas de control de infecciones en unidades neonatales para contener la diseminación de cepas multidrogorresistentes y mejorar la supervivencia neonatal.

## EL ESTUDIO

### Diseño y ámbito de estudio

Estudio transversal descriptivo realizado en el servicio de Neonatología del HNGAI durante el periodo 2020-2024. Este hospital es un establecimiento de alta complejidad del Seguro Social y es un centro de referencia nacional ubicado en Lima, Perú.

### Participantes

La población incluyó neonatos hospitalizados en el área de neonatología o la Unidad de Cuidados Intensivos Neonatales (1 de enero de 2020 - 31 de diciembre de 2024), con hemocultivo monomicrobiano positivo a KpPC dentro de los primeros 28 días de vida. La clasificación en bacteriemia primaria o secundaria se basó en el origen clínico de la infección y no en el tipo de muestra microbiológica. El flujograma de selección se muestra en la figura 1.

**Figura 1:** Flujograma de neonatos incluidos y excluidos con hemocultivo positivo para *Klebsiella pneumoniae* productora de carbapenemasas.

^a^ El análisis microbiológico y de susceptibilidad antimicrobiana presentado en el estudio corresponde exclusiva-mente a las cepas de los pacientes incluidos (n=19).

### Variables de estudio

Registramos el sexo, el peso al nacer (en gramos), la edad gestacional (en semanas completas), el tipo de parto, las fechas de nacimiento, toma del hemocultivo y egreso hospitalario. La edad al momento de la bacteriemia fue calculada en días como la diferencia entre la fecha de toma de hemocultivo y de nacimiento. La prematuridad fue definida como el nacimiento antes de las 37 semanas de gestación, y el bajo peso al nacer como < 2500 gramos. Un especialista en infectología (GPL) clasificó las bacteriemias como primarias o secundarias a partir de las historias clínicas.

El tiempo total de estancia hospitalaria en días fue calculado como la diferencia entre la fecha del desenlace (vivo o fallecido) y el ingreso. También se determinó el tiempo de estancia hasta el aislamiento de KpPC (en días) y el tiempo hasta la administración de la terapia antibiótica dirigida apropiada (en horas), determinado por la susceptibilidad *in vitro* de al menos un antibiótico ^12^^,^^13^. Se registró el perfil de susceptibilidad, la terapia empírica administrada, la adecuación de dicha terapia en base a los resultados del antibiograma y el tratamiento definitivo de cada neonato.

### Procedimientos microbiológicos

Los datos microbiológicos fueron solicitados al servicio de microbiología del HNGAI y obtenidos de las historias clínicas electrónicas. En el HNGAI la identificación inicial de los hemocultivos se realizó por microbiología convencional. La susceptibilidad antimicrobiana fue realizada mediante la plataforma VITEK-2® GN y la confirmación de carbapenemasas mediante métodos inmunocromatográficos (RESIST-5 O.K.N.V., Coris Bio-Concept) y Xpert ® Carba (Cepheid). La evaluación de la susceptibilidad a colistina se realizó mediante Colistin agar spot testagar. La concentración mínima inhibitoria de cada antibiótico siguió las pautas establecidas por el Instituto de Estándares Clínicos y de Laboratorio (CLSI). Para colistina, se utilizaron los puntos de corte CLSI vigentes (M100), que consideran únicamente las categorías: intermedia (≤2 µg/mL) y resistente (≥4 µg/mL) para Enterobacterales ^14^.

### Análisis estadístico

Reportamos las medidas de tendencia central (mediana, rango intercuartílico [RIC]) para las variables cuantitativas, y la frecuencia para las variables cualitativas. El análisis descriptivo fue realizado con el programa Jamovi (versión 2.3.28).

### Aspectos éticos

El protocolo fue aprobado por el Comité de Ética de la Universidad de Piura (Nº T0624-11) y el Comité Institucional de Ética en Investigación del HNGAI (Nº 108-2024).

## HALLAZGOS

De un total de 40 hemocultivos con aislamiento de KpPC identificados durante el periodo de estudio, 19 correspondieron a neonatos dentro de los primeros 28 días de vida, de acuerdo con la población definida en el estudio, siendo estos los casos finalmente analizados (figura 1). Todas fueron cepas productoras de NDM, no se identificaron otras carbapenemasas. El 52,6% fueron mujeres, 94,7% fueron prematuros y todos tuvieron bajo peso al nacer. La mediana de edad gestacional fue de 27 semanas (RIC: 26 - 30). El 94,7% (18/19) tuvo bacteriemia secundaria. La terapia de soporte incluyó el uso de sonda orogástrica y catéter central en los 19 casos, nutrición parenteral en 18 (94,7%) y ventilación mecánica en 15 casos (78,9%). Todos los neonatos tuvieron algún tipo de alteración metabólica. El 38,9% de los neonatos (7/19) tuvieron dos alteraciones metabólicas simultáneamente. El 84,2% (16/19) de neonatos presentaron cuatro o más comorbilidades (tabla 1).

**Tabla 1:** Características clínicas, soporte terapéutico y comorbilidades de neonatos con bacteriemia por *Klebsiella pneumoniae* productora de NDM (n=19).

Variables		Frecuencia absoluta (n=19)	Porcentaje (%)^b^
Sexo			
	Femenino	10	52,6
	Masculino	9	47,4
Tipo de parto			
	Natural	2	10,5
	Cesárea	17	89,5
	Prematuridad (sí)	18	94,7
	Peso al nacer (bajo<2500 g)	19	100,0
Área al momento del diagnóstico			
	UCI	15	78,9
	Hospitalización	4	21,1
Tipo de bacteriemia			
	Primaria	1	5,3
	Secundaria	18	94,7
Foco de infección^a^			
	Origen respiratorio	9	50,0
	Origen gastrointestinal	7	38,9
	Relacionado a catéter	2	11,1
Terapia de soporte^b^			
	Sonda orogástrica	19	100,0
	Catéter central	19	100,0
	Nutrición parenteral total	18	94,7
	Ventilación mecánica	15	78,9
	Uso de surfactante	11	57,9
	Uso de vasopresores	8	42,1
	Uso de corticoides	6	31,6
	Catéter urinario	5	26,3
	Cirugías	1	5,3
	Catéter periférico	1	5,3
Comorbilidades			
	Alteraciones metabólicas^c^	19	100,0
	Distrés respiratorio	17	89,5
	Enfermedad de membrana hialina	14	73,7
	Anemia	11	57,9
	Hiperglicemia	10	52,6
	Hipoalbuminemia	6	31,6
	Hipoglicemia	6	31,6
	Cardiopatía congénita	4	21,1
	Falla renal	1	5,3
	Otra comorbilidad^d^	14	73,7
Número de comorbilidades por neonato			
	Una	0	0,0
	Dos	1	5,3
	Tres	2	10,5
	Cuatro o más	16	84,2
Condición final			
	Vivo	15	78,9
	Fallecido	4	21,1

UCI: unidad de cuidados intensivos; g: gramos.

a El foco de infección se describe únicamente en los casos de bacteriemia secundaria (n=18); los porcentajes se calcularon sobre este subgrupo.

b El porcentaje es calculado considerando como denominador los 19 neonatos.

c Acidosis metabólica 47,4% (9/19); hipocalcemia: 47,4% (9/19); hiponatremia: 42,1% (8/19); acidosis respiratoria: 21,1% (4/19); hipernatremia: 15,8% (3/19).

d Ictericia neonatal: 57,1% (8/14); corioamnionitis: 14,3% (2/14); enterocolitis necrotizante: 7,1% (1/14).

La mediana del tiempo de estancia hospitalaria fue de 82 días (RIC: 30,5 - 92,5), la mediana del tiempo hasta la toma de hemocultivo fue de 9 días (RIC: 7,50 - 15,5) y la mediana de tiempo hasta el inicio de terapia antibiótica dirigida apropiada fue de 94,5 horas (RIC: 48,3 - 103). Cuatro de los 19 neonatos fallecieron durante toda la hospitalización (21,1%), de los cuales tres fallecieron en los primeros 28 días de nacimiento (15,8%). En estos últimos, la mediana de días desde la bacteriemia hasta la muerte fue de 2 (RIC: 1,5 - 6,5).

 El tratamiento antimicrobiano empírico incluyó regímenes con piperacilina/tazobactam en 36,8% (7/19) y vancomicina en 57,9% (11/19). Las combinaciones utilizadas empíricamente fueron piperacilina/tazobactam y vancomicina en 26,3% (5/19), seguida de ceftazidima y amikacina en 11,1% (2/19). El tratamiento empírico fue apropiado en 7 de 16 casos (43,8%), además, no fue posible definir la adecuación en tres casos donde se utilizó ampicilina, oxacilina y gentamicina debido a que no fueron evaluados en los antibiogramas. Hubo susceptibilidad conservada a colistina, amikacina y tigeciclina en 100% (19/19), 84,2% (16/19) y 40% (6/15) de los aislamientos, respectivamente. Se observó resistencia a ampicilina/sulbactam, ceftazidima, ciprofloxacino, meropenem, imipenem y piperacilina/tazobactam en todos los casos. La terapia antimicrobiana dirigida se administró en 18 neonatos; de estos, 16 (88,9%) recibieron esquemas combinados, siendo la combinación de amikacina, colistina y tigeciclina la más frecuente (figura 2).

**Figura 2:** Terapia antibiótica individual empírica y definitiva y perfil de resistencia de neonatos con bacteriemia por *K. pneumoniae* productora de NDM.

S: sensible; I: intermedio; R: resistente; ATM: aztreonam; AMK: amikacina; AMC: amoxicilina/ácido clavulánico; AMP: ampicilina; SAM: ampicilina/ sulbactam; ATM; aztreonam; FEP: cefepime; CTX: cefotaxima; C/T: ceftolozano/tazobactam; CAZ: ceftazidima; CZA: ceftazidima/avibactam; CXM: cefuroxima; CIP: ciprofloxacino; CST: colistina; ETP: ertape-nem; GEN: gentamicina; IPM: imipenem; MEM: meropenem; TGC: tigeciclina; SXT: trimetoprim/sulfametoxazol; OXA: oxacilina; VAN: vancomicina; ZTP: piperacilina/tazobactam. ^a^ No fue posible determinar la adecuaciónde la terapia empírica en los casos 3, 8 y 11 debido a la falta de evaluación de ampicilina, oxacilina y gentamicina en los respectivos antibiogramas.

## DISCUSIÓN

Encontramos que todas las cepas de KpPC aisladas fueron productoras de NDM. Clínicamente los neonatos fueron pretérminos y de bajo peso al nacer. El foco de infección en la mitad de los casos fue respiratorio. A nivel microbiológico, las cepas fueron sensibles a amikacina y tigeciclina, y tuvieron susceptibilidad intermedia a colistina, mientras que todas las cepas fueron resistentes a ampicilina/sulbactam, ceftazidima, ciprofloxacino, meropenem, imipenem y piperacilina/tazobactam. Con dicho perfil, el tratamiento empírico fue apropiado en siete de dieciséis neonatos, y una quinta parte de los neonatos fallecieron.

En nuestra muestra sólo se identificó el gen *bla*_NDM_, carbapenemasa dominante en 14 países alrededor del mundo ^6^. Sin embargo, ciertas regiones muestran perfiles distintos, siendo KPC-2 el tipo más frecuente en un hospital pediátrico de Brasil, mientras que en una cohorte española de niños predominó VIM ^9^^,^^15^.

Con relación al perfil de susceptibilidad, encontramos similitud con un estudio en neonatos de un hospital en China, en quienes se encontró KpRC con sensibilidad a colistina y tigeciclina en la mayoría de los aislamientos; sin embargo, a diferencia de nuestro estudio, la mayoría de neonatos presentaron resistencia a amikacina ^16^. Asimismo, la resistencia a tigeciclina en nuestro estudio (60%) fue mayor que la reportada en niños menores de 5 años con sepsis por *K. pneumoniae* en Etiopía (39%) ^17^. Este hallazgo resalta la relevancia global de este perfil de resistencia y la influencia de factores epidemiológicos en su variabilidad.

El uso de colistina y tigeciclina como tratamiento definitivo combinado fue frecuente en la mayoría de los neonatos; sin embargo, los regímenes óptimos para dichos antibióticos en esta población tienen evidencia limitada ^18^. La terapia empírica fue apropiada en 43,8% de los neonatos, lo cual puede ser resultado de la heterogeneidad en los regímenes de tratamiento brindados. El tiempo desde la toma del hemocultivo hasta el inicio de la terapia dirigida tuvo una mediana de 95 horas, lo cual refleja las dificultades para optimizar tempranamente la terapia antibiótica contra NDM y podría verse relacionado con la mortalidad encontrada en nuestro estudio. La evidencia muestra que la terapia dirigida apropiada reduce en un 67% la mortalidad en niños con bacteriemia por KpRC ^16^. Además, iniciar oportunamente un antibiótico con actividad confirmada *in vitro* disminuye en un 80% la mortalidad a los 30 días ^15^.

La mortalidad en nuestro estudio fue ~21%, este resultado es consistente con lo estimado en una revisión sistemática para KpRC neonatal (mortalidad agrupada ~23%), en este estudio la combinación de alta vulnerabilidad del huésped y ventanas terapéuticas tardías fueron determinantes del desenlace ^6^. Sin embargo, en un estudio en China reportaron una mayor mortalidad en neonatos con bacteriemia por KpRC (39,4%) ^16^. La menor mortalidad en nuestro estudio podría relacionarse a la susceptibilidad conservada de los antimicrobianos como colistina y amikacina, al igual que la gravedad de los casos.

Además, la mortalidad observada podría explicarse por las características clínicas de nuestros neonatos: prematuridad, bajo peso al nacer, soporte invasivo múltiple, focos respiratorios o gastrointestinales, y hospitalización prolongada. Estos hallazgos son consistentes con estudios en China y Egipto, donde la prematuridad, el bajo peso y el uso de dispositivos invasivos, incluida la nutrición parenteral total, se asociaron con un mayor riesgo de infección por Enterobacterales resistentes a carbapenémicos (ERC) ^19^^,^^20^.

Los focos de infección más frecuentes fueron el respiratorio, seguido del gastrointestinal. Dichos resultados contrastan con una revisión sistemática realizada en Latinoamérica en pacientes pediátricos con bacteriemia por ERC, quienes reportaron que el 64% de las bacteriemias por EPC fueron secundarias a foco intraabdominal, seguidas por focos cutáneos en 32% ^21^. Este contraste se puede deber a la alta frecuencia de ventilación mecánica en nuestra población (78%).

El tiempo de estancia prolongada refleja mayor riesgo de infecciones nosocomiales y comorbilidades. En nuestro estudio, la mediana de estancia hospitalaria fue de 82 días, superior a lo reportado en Italia donde se documentó una mediana de 70 días entre neonatos con bacteriemia por KP multirresistente ^22^, mientras que en China se registró 36 días en neonatos con bacteriemia por KpRC ^16^. Esta diferencia podría reflejar un diagnóstico más precoz o una progresión más rápida de la infección en nuestra población. Además, reportamos una mediana de edad hasta la bacteriemia de 9 días, lo que resulta menor a lo reportado en Portugal y Brasil que fue de 12 días en casos de colonización de EPC ^7^^,^^9^. Estos hallazgos sugieren una posible colonización más temprana en nuestra muestra que podría deberse a factores individuales y del entorno hospitalario.

Entre las limitaciones, este estudio fue retrospectivo y tuvo una muestra reducida; sin embargo, recopilamos información de todos los neonatos con bacteriemia por KpPC tipo NDM atendidos bajo condiciones habituales de un mismo establecimiento de referencia. Además, la definición de terapia antimicrobiana empírica apropiada pudo verse afectada por paneles de susceptibilidad parciales; a pesar de ello, describimos su adecuación en 16 neonatos tratados empíricamente con antibióticos y evaluados con el perfil de susceptibilidad. Asimismo, los esquemas terapéuticos observados reflejan la práctica clínica en un hospital público de referencia y estuvieron condicionados por la disponibilidad local de antimicrobianos. En infecciones por enterobacterias productoras de metalo-β-lactamasas, esquemas como la combinación de ceftazidima-avibactam más aztreonam han generado interés; sin embargo, su uso en neonatos aún carece de evidencia clínica robusta. Además, el uso de ceftazidima-avibactam en neonatos ha sido históricamente fuera de etiqueta, dado que su aprobación regulatoria inicial se restringió a pacientes mayores de 3 meses ^23^^,^^24^.

Otra limitación fue la ausencia de estudios de tipificación molecular o pruebas de clonalidad de los aislamientos. Si bien todas las cepas fueron productoras de NDM, no se realizó la relación clonal mediante alguna técnica de secuenciación genómica. Por ello, no es posible determinar si los casos corresponden a la diseminación de un mismo clon dentro de la unidad neonatal o a múltiples introducciones independientes. Esta información sería relevante para comprender mejor la dinámica de transmisión y orientar estrategias de control de infecciones.

En conclusión, KpPC tipo NDM predominó en neonatos de bajo peso al nacer y prematuros del servicio de UCI neonatal. Además, observamos una mayor frecuencia de bacteriemia secundaria con foco respiratorio. Este estudio evidenció que la *Klebsiella pneumoniae* productora de NDM fue resistente a múltiples fármacos, sin embargo, fue susceptible a amikacina y colistina. La identificación de *bla*_NDM_ refuerza la importancia de la vigilancia genómica local para orientar la terapia empírica. El tipo de carbapenemasa y su impacto en la mortalidad representan una brecha de investigación futura.
